# The Relation Between Supervisors’ Big Five Personality Traits and Employees’ Experiences of Abusive Supervision

**DOI:** 10.3389/fpsyg.2016.00112

**Published:** 2016-02-10

**Authors:** Jeroen Camps, Jeroen Stouten, Martin Euwema

**Affiliations:** ^1^Organisational Leadership & Decision-Making Group, Cambridge Judge Business School, University of CambridgeCambridge, UK; ^2^Research Group Occupational & Organisational Psychology and Professional Learning, KU LeuvenLeuven, Belgium

**Keywords:** abusive supervision, FFM, Big Five personality traits, leadership, perceived supervisor mistreatment

## Abstract

The present study investigates the relation between supervisors’ personality traits and employees’ experiences of supervisory abuse, an area that – to date – remained largely unexplored in previous research. Field data collected from 103 supervisor-subordinate dyads showed that contrary to our expectations supervisors’ agreeableness and neuroticism were not significantly related to abusive supervision, nor were supervisors’ extraversion or openness to experience. Interestingly, however, our findings revealed a positive relation between supervisors’ conscientiousness and abusive supervision. That is, supervisors high in conscientiousness were more likely to be perceived as an abusive supervisor by their employees. Overall, our findings do suggest that supervisors’ Big Five personality traits explain only a limited amount of the variability in employees’ experiences of abusive supervision.

Let me tell you something. You stupid little runt. I own you. You’re my bitch. So don’t walk around here thinking you have free will, because you don’t. I could crush you anytime I want. So settle in, cause you are here for the long haul.

– Kevin Spacey in Horrible Bosses

## Introduction

In this passage from the movie Horrible Bosses, Dave Harken (played by Kevin Spacey) addresses an employee in an utmost rude manner and thereby demonstrates to the viewer what it is like to work for an *abusive supervisor*. Abusive supervision, defined as “subordinates’ perceptions of the extent to which supervisors engage in the sustained display of hostile verbal and non-verbal behaviors, excluding physical contact” (Tepper, 2000, p. 178), has been shown to result in a wide variety of harmful consequences for targeted employees (e.g., lower levels of job and life satisfaction; see Tepper, 2000), their coworkers (e.g., in terms of experienced interpersonal mistreatment; see Mitchell and Ambrose, 2007), their organization (e.g., lower levels of employee performance; see Harris et al., 2007) and even their family members (e.g., in terms of family undermining; see Hoobler and Brass, 2006). As a result, abusive supervision not only harms targeted employees and their peers, but also relates to the success and survival of the organizations themselves (Martinko et al., 2013). Therefore, rendering insights on antecedents of abusive supervision is highly valuable.

Previous research revealed that, abusive supervision can be partially explained by, for example, mistreatment by the supervisor’s own manager (Bardes Mawritz et al., 2012), workplace stress (Burton et al., 2012), low performance levels of their subordinates (Tepper et al., 2011b) and how supervisors were treated by their parents during childhood (Kiewitz et al., 2012). While these studies have clearly advanced our understanding of how situational factors – and *interpersonal* relations – contribute to the prevalence of experienced supervisory abuse, *intrapersonal* theorizing (e.g., personality) on abusive supervision is still underdeveloped. Part of the little research that did focus on supervisor personality, Kiazad et al. (2010) showed that supervisors’ Machiavellianism is relevant for predicting abusive supervision. Yet, the global *supervisor dispositions* that contribute to abusive supervision have not been examined. This is surprising, especially, since previous studies suggested that organizations should adopt a ‘no-hire policy’ for supervisors who have the *potential* to turn to abusive supervision (e.g., Burton and Hoobler, 2011; Tepper et al., 2011b; Chi and Liang, 2013). We address this issue by exploring the Five Factor Model (FFM) of personality (Costa and McCrae, 1992) in the prevalence of abusive supervision.

The present study contributes to the theorizing on the antecedents of abusive supervision by focusing on intrapersonal factors and more specifically, drawing on the FFM of personality (Goldberg, 1990). That is, our study explores the influence of supervisors’ agreeableness, conscientiousness, extraversion, neuroticism, and openness to experience on employees’ experiences of abusive supervision. Below, we elaborate on the theoretical foundations of our study.

### Antecedents of Abusive Supervision

Even though knowledge on the consequences of abusive supervision still largely outweighs what is known about its antecedents (Martinko et al., 2013), research has started to shift its focus toward exploring which factors stimulate or inhibit the prevalence of abusive supervision (Zhang and Bednall, 2015). Several of these studies have explained abusive supervision in terms of displaced aggression. For example, employees report higher levels of abusive supervision when their supervisor experiences workplace stress (Burton et al., 2012), procedural/interactional injustice (see, respectively, Tepper et al., 2006; Aryee et al., 2007), psychological contract breach (Hoobler and Brass, 2006) or when their supervisor was exposed to family undermining during childhood (Kiewitz et al., 2012). Recently, research has started to acknowledge that employees might also (unwillingly) foster the prevalence of abusive supervision. Amongst others, studies have established a link between employees’ personality (Brees et al., 2014; Henle and Gross, 2014), performance (Tepper et al., 2011b) as well as organizational deviance (Lian et al., 2014) and abusive supervision. Whilst research on the influence of situational and interpersonal factors in the prevalence of abusive supervision has steadily grown, studies exploring the importance of supervisor dispositions in the prevalence of abusive supervision are surprisingly scarce. The only studies – to our knowledge – that have pursued this direction revealed that supervisors’ Machiavellianism is positively related to abusive supervision (Kiazad et al., 2010), while supervisors’ Emotional Intelligence is negatively related to abusive supervision (Xiaqi et al., 2012). Even though specific personality traits are relevant, it is also important to address conglomerate dimensions of personality, such as the Big Five-factor model (being the most commonly used personality framework), and their relations to abusive supervision. Currently, no studies on the relation between supervisors’ Big Five personality and perceived abusive supervision have been conducted (Einarsen et al., 2013). In response to this, the present study explores the role of supervisors’ Big Five personality in the prevalence of employees’ experiences of abusive supervision.

### Supervisors’ Big Five Personality and Abusive Supervision

The Big Five personality model is probably the most widely used framework in personality research as well as in practice. Even though previous research has demonstrated its utility in predicting leadership, such as transformational leadership (Bono and Judge, 2004) and ethical leadership (Kalshoven et al., 2011), no research – to our knowledge – has explored its predictive value regarding employees’ experiences of abusive supervision. Given the absence of such insights, we draw from more general personality research as well as a wide variety of studies linking supervisors’ Big Five personality to leadership practices (see also Judge and Long, 2012), such as transformational leadership (e.g., Bono and Judge, 2004), ethical leadership (e.g., Kalshoven et al., 2011), and leadership emergence/effectiveness (e.g., Judge et al., 2002a), in order to explore how each Big Five personality trait might relate to abusive supervision. Below, we will elaborate on each of the Big Five personality traits (i.e., agreeableness, conscientiousness, extraversion, neuroticism, and openness to experience) and explain for each personality trait, how it might be relevant to the prevalence of abusive supervision.

#### Agreeableness

People who are high in agreeableness are generally speaking kind, considerate, and helpful toward others. Agreeable persons aim to keep relationships harmonious and have a preference for the use of compromises in dealing with conflicts (e.g., Graziano et al., 1996). People who are more agreeable are highly cooperative, and sociable (Skarlicki et al., 1999), and are more likely to regulate their angry feelings (Jensen-Campbell et al., 2003). Low agreeable persons, by contrast, seem to lack a concern for others’ welfare and are less capable of inhibiting aggressive responses (see Costa and McCrae, 1992; Judge et al., 2013). Abusive supervision covers a variety of these responses, such as a supervisor ridiculing an employee in front of others and shifting the blame in order to save him/herself (see Tepper, 2000). Given the harmful consequences for those who experience supervisory abuse, supervisors who are high in agreeableness (and thus particularly oriented toward fostering the wellbeing of their employees) should be more likely to refrain from abusive supervision. In support of this assumption, previous research by Mayer et al. (2007) revealed that supervisors high in agreeableness indeed create a working climate in which interpersonal fair treatment is highly valued. Finally, Tepper (2007, p. 281) also suggested that “supervisors who are low in agreeableness should be relatively unconcerned about the effects their behavior may have on the quality of relationships with subordinates (i.e., the possibility that their behavior might be perceived as argumentative, hostile, and conflictive; Costa et al., 1989) and should therefore be more likely to behave abusively toward subordinates compared to supervisors who are higher in agreeableness.” Following these arguments, we expect the following:

*Hypothesis 1*: Supervisors’ agreeableness is negatively related to abusive supervision.

#### Conscientiousness

Conscientiousness is characterized by being hardworking, persistent, neat, well-organized and goal-oriented (Costa and McCrae, 1992). Several studies have highlighted its benefits in terms of, for example, increased performance (Judge et al., 2013), the use of more effective coping strategies (Carver and Connor-Smith, 2010), as well as lower levels of interpersonal deviance (e.g., O’Neill et al., 2011). Moreover, supervisors who are high in conscientiousness tend to create a fair environment for their employees (Mayer et al., 2007) and are often seen as demonstrating ethical behavior (Kalshoven et al., 2011), which suggest that these supervisors should be less likely to be perceived as being abusive. Yet, even though conscientiousness has a variety of desirable consequences, recently, studies have revealed that conscientiousness might also have a more negative side. Conscientious supervisors’ strong orientation toward the achievement of goals might result in micro-managing employees and being perceived as difficult to please (Judge and Long, 2012). Relatedly, research on abusive supervision revealed that employees sometimes report that their supervisor engages in abusive supervision due to performance-driven motives (Liu et al., 2012). Abusive supervision can indeed emerge as a response to poor employee performance, particularly when supervisors’ own outcomes are dependent on their subordinates’ performance (Walter et al., 2015). In line with these findings Tepper et al. (2011a) suggested that supervisors might (deliberately) use abusive supervision as an influence tactic in their pursuit of personal or unit performance objectives. Following this rationale and given that high conscientious individuals are strongly oriented toward goal-achievement, employees working for such supervisors could thus be more at risk to experience (or perceive) abusive supervision. In sum, given the mixed arguments regarding whether supervisors’ conscientiousness stimulates versus inhibits perceived abusive supervision, we propose no specific hypothesis regarding its relation with employees’ experiences of supervisory abuse.

#### Extraversion

Extraverts are known for being sociable and assertive, as well as for their tendency to experience positive emotions (Costa and McCrae, 1992). They are enthusiastic, seek excitement and are more likely to emerge into a supervisory position (Costa and McCrae, 1992; Judge et al., 2002a). However, theory is less straightforward to whether they are more or less likely to turn to abusive supervision. For example, of the Big Five personality traits, extraversion consistently predicts leadership effectiveness (Judge et al., 2002a). Abusive supervision, however, has been linked to *lower* leadership effectiveness as it results in diminished performance and increased resistance behavior on the part of targeted employees (Tepper, 2007). Following these findings, supervisors who are high in extraversion should be less likely to display abusive supervision. Similarly, extraverts prefer the company of others and tend to be friendly and warm in their interactions (Costa and McCrae, 1992), which should make them less likely to be perceived as an abusive supervisor. Conversely, however, interactions with extraverts (as compared to introverts) have been linked to experienced relation conflict (Bono et al., 2002), which suggests that extraverted supervisors are more likely to develop a conflictual relationship with their employees (Judge and Long, 2012). Relatedly, extraverts’ tendency to be bold in their communication toward others (Judge and Long, 2012), might cause employees working for an extraverted supervisor to experience his/her verbal communication as abusive. Given the mixed arguments for the relation between supervisors’ extraversion and abusive supervision, we propose no specific hypothesis regarding its influence on employees’ experiences of supervisory abuse.

#### Neuroticism

Neuroticism (sometimes also labeled as its opposite, Emotional Stability) captures one’s tendency to experience a variety of disruptive emotions and thoughts. People who are high in neuroticism are insecure, anxious and are more susceptible to stress than their low-neurotic counterparts (Costa and McCrae, 1992). They experience higher levels of negative affect, get easily irritated by others, and are more likely to turn to inappropriate coping responses, such as interpersonal hostility (see for example McCrae and Costa, 1987; Judge et al., 2013). As such, supervisors’ neuroticism should be relevant toward predicting abusive supervision, particularly because previous research has already established that higher levels of stress (Bardes Mawritz et al., 2012) and negative emotions (Hoobler and Hu, 2013) are indeed positively related to instances of supervisory abuse. In line with these arguments, Tepper (2007, p. 281) proposed that “supervisors who are high in neuroticism experience greater anger, frustration, and impulsiveness compared with their low-neuroticism counterparts (Costa and McCrae, 1992), and consequently, neuroticism should be positively related to abusive supervision.” As such, we postulate the following:

*Hypothesis 2*: Supervisors’ neuroticism is positively related to employees’ experiences of abusive supervision.

#### Openness to Experience

Openness to experience is characterized by a tendency to have an active imagination, an intellectual curiosity as well as the willingness to consider new ideas and try new things (Costa and McCrae, 1992). As a result, individuals high in openness are more receptive to input from others and are less authoritarian (McCrae and Sutin, 2009), which has been shown to inhibit the emergence of abusive supervision (Kiazad et al., 2010). A study by Stewart et al. (2005), however, also revealed that team members high in openness to experience are perceived to be less cooperative and friendly. Interestingly, Caprara et al. (1996) revealed individuals’ openness to experience to be negatively correlated with their irritability and hostility, but also positively correlated with their tendency to engage in verbal aggression. Moreover, they concluded that openness (as well as conscientiousness) contributes little toward understanding individual differences in aggressive tendencies. Based on these findings and in line with the fact that Tepper (2007) did not describe about a relation between supervisors’ openness and abusive supervision, we propose no specific hypothesis and investigate the influence of supervisors’ openness to experience from an exploratory perspective.

## Materials and Methods

### Participants and Procedure

We solicited participation for a study on leadership and decision making from a variety of organizations in our research network. When a person declaring his/her interest to participate held a supervisory position, we asked him/her to provide us with the contact information of all of his/her employees, so that we could randomly select one of these employees to fill out the focal employee questionnaire. Conversely, employees who did not hold a supervisory position were asked to provide the contact information of their direct supervisor. All participants were informed that participation would take approximately 15 min of their time and received a personalized e-mail containing both a link to the online questionnaire and a personal code that allowed us to match the answers of the supervisors with those of the corresponding focal employees. They were guaranteed that their answers would be processed anonymously. Following this procedure, we obtained data from 115 supervisors and 110 focal employees. Overall, this resulted in matched data of 103 supervisor-focal employee dyads from a variety of industries, such as health care, government, and the banking industry.

The study was carried out in line with the recommendations of the Social and Societal Ethics Committee of the University of Leuven (research ID: G-2014 08 037). Employees and supervisors who followed the link to the online questionnaire were presented with (an electronic version of) the Informed Consent. Amongst other things, the Informed Consent mentioned that participation in the Study was voluntarily and that participants had the right to end the study at any time without any negative consequence. At the end of Informed Consent participants were explicitly asked to indicate their agreement with the information presented. We constructed the online questionnaire so that participants who did not indicate their agreement would have been automatically guided to the ending screen and thanked for their interest. All participants, however, provided Informed Consent.

The variables of interest were obtained from different sources in order to reduce common source bias (Podsakoff et al., 2003). More specifically, focal employees were asked to rate the extent to which they perceived their supervisor to engage in abusive supervision, while supervisors were asked to complete the Big Five personality measure. Supervisors had an average age of 45.04 years (*SD* = 7.75), 97.09% worked on a fulltime basis and 38.83% were female. Of these supervisors, 1.94% were blue-collar workers, 92.23% were white-collar workers and 5.83% were self-employed. They had an average organizational tenure of 15.51 years (*SD* = 8.41); 4.85% completed only secondary school, 26.21% obtained a bachelor’s degree, 50.49% obtained a master’s degree, and 18.45% obtained a postgraduate degree or Ph.D. The focal employees were on average 40.57 years old (*SD* = 8.62), 53.40% were female and 87.38% worked on a fulltime basis. Of these participants, 97.09% were white-collar workers, 1.94% were blue-collar workers and 0.97% were self-employed. They had an average organizational tenure of 11.56 years (*SD* = 8.56); 10.68% completed only secondary school, 44.66% obtained a bachelor’s degree, 36.89% obtained a master’s degree, and 6.80% obtained a postgraduate degree or Ph.D. (0.97% failed to indicate their highest education).

Finally, we explored whether employees and participants differed in terms of these demographics. One-way analyses of variance revealed that the focal employee sample and supervisor sample did not significantly differ in terms of whether they were white-collar workers, blue-collar workers, or self-employed [*F*(1,204) = 2.29, *p* = 0.13]. However, focal employees and supervisors did significantly differ in terms of age [*F*(1,204) = 15.29, *p* < 0.001], gender [*F*(1,204) = 4.45, *p* < 0.05], organizational tenure [*F*(1,204) = 11.17, *p* = 0.001], their educational level [*F*(1,203) = 15.11, *p* < 0.001] and whether they worked part-time or full-time [*F*(1,204) = 6.94, *p* < 0.01]. **Table 1** provides a detailed overview of the specific demographics of each group.

**Table 1:** Demographic information.

	Employees	Supervisors
Average age	40.57	45.04
Gender (% Female)	53.40	38.83
Fulltime (%)	87.38	97.09
Occupational classification		
Blue-collar workers (%)	1.94	1.94
White-collar workers (%)	97.09	92.23
Self-employed (%)	0.97	5.83
Average organizational tenure	11.56	15.51
Education		
Secondary school (%)	10.68	4.85
Bachelor’s degree (%)	44.66	26.21
Master’s degree (%)	36.89	50.49
Postgraduate degree or Ph.D. (%)	6.80	18.45


### Measures

Given that our sample consisted of both Dutch-speaking and English-speaking employees and supervisors we developed two different versions of the questionnaires. The English questionnaire consisted of the original scales, while the Dutch questionnaire was constructed following a careful translation-back-translation procedure.

#### Big Five Personality Traits

We measured supervisors’ personality with the 44-item Big Five Inventory (BFI; John et al., 1991). The BFI consists of 44 items and was developed in order to provide researchers with a shorter, yet reliable, instrument for assessing the Big Five personality traits. The validity and reliability of the BFI in light of the NEO-FFI has been explored and confirmed by John and Srivastava (1999). Since its development the BFI has been used in variety of studies for the purpose of assessing participants’ broad Big Five personality traits (see for example Middleton, 2005; Judge et al., 2006). Agreeableness (A) was measured with nine items (α = 0.79), conscientiousness (C) was measured with nine items (α = 0.76), extraversion (E) was measured with eight items (α = 0.85), openness to experience (O) was measured with 10 items (α = 0.74), and neuroticism (N) was measured with eight items (α = 0.83). All items were scored using a 5-point scale ranging from 1 (disagree strongly) to 5 (agree strongly). Sample items are “I see myself as someone who is talkative (E),” “I see myself as someone who tends to find fault with others (A),” “I see myself as someone who does a thorough job (C),” “I see myself as someone who is depressed, blue (N),” and “I see myself as someone who is original, comes up with new ideas (O).” For each trait we averaged the item scores in order to obtain total scores for each trait.

#### Abusive Supervision

We measured abusive supervision (α = 0.69) with the 5-item measure developed by Mitchell and Ambrose (2007) based on Tepper’s (2000) original measure. This measure captures employees’ perceptions of their supervisor’s active interpersonal abuse and was validated by Mitchell and Ambrose (2007; see Appendices A and B in their manuscript for the EFA and CFA results) with the data from Tepper (2000) and Tepper et al. (2004). This measure has been used in multiple studies (e.g., Thau and Mitchell, 2010; Lin et al., 2013) to measure abusive supervision and has been shown to yield similar results as Tepper’s (2000) original measure (see Tepper et al., 2008; Decoster et al., 2014). Employees indicated on a 7-point scale ranging from 1 (totally not agree) to 7 (totally agree) to what extent they agreed with the following statements “My supervisor ridicules me,” “My supervisor makes negative comments about me to others,” “My supervisor tells me my thoughts or feelings are stupid,” “My supervisor tells me I’m incompetent,” and “My supervisor puts me down in front of others.” The item scores were averaged to create total scores reflecting participants’ perceptions of abusive supervision.

#### Control Variables

We opted to control for supervisors’ organizational tenure, age and gender because previous research revealed that these demographics might be related to experienced interpersonal mistreatment (Gilbert and Tang, 1998; Aquino and Douglas, 2003; Henle et al., 2005). However, given that of these three variables only supervisors’ age was correlated with abusive supervision, we followed Becker’s (2005) suggestion and did not include supervisors’ gender and organizational tenure as control variables in our regression analyses. In order to fully inform the readers, **Table 3** contains the regression analyses both with and without this control variable. Finally, although we did not expect the outcome of our analyses to be affected by whether supervisors and employees filled out a Dutch version or an English version of the questionnaire, we decided to run additional analyses to ensure that this indeed was not the case. In line with Becker’s (2005) suggestions, we first investigated whether the language of the questionnaire that supervisors and employees filled out was significantly related to abusive supervision. Simple correlations revealed that abusive supervision was significantly correlated with the language of the supervisor questionnaire, but not with the language of the employee questionnaire (see **Table 2**). As such, we followed Becker’s (2005) suggestions and retested our hypotheses while we included the language of the supervisor questionnaire as an additional control variable. The results presented at the right side of **Table 3** reveal that including this additional control variable does not affect the outcome of our Hypotheses tests, nor the interpretation of our findings.

**Table 2:** Descriptive statistics and intercorrelations.

		*Mean*	*SD*	1	2	3	4	5	6	7	8	9	10	11
1	Supervisor age	45.04	7.75	–
2	Supervisor gender	0.39	0.49	-0.07	–
3	Supervisor organizational tenure	15.51	8.41	0.40ˆ***	-0.03	–
4	Language supervisor Questionnaire	0.65	0.48	-0.05	0.13	-0.07	–
5	Language employee Questionnaire	0.66	0.48	-0.05	0.15	-0.02	0.94ˆ***	–
6	Agreeableness	4.09	0.51	0.10	0.27ˆ**	0.07	-0.08	-0.10	–
7	Conscientiousness	4.09	0.50	0.02	0.21ˆ*	0.14	-0.30ˆ**	-0.26ˆ**	0.43ˆ***	–
8	Extraversion	3.93	0.65	-0.02	0.05	-0.16	0.09	0.07	0.37ˆ***	0.11	–			
9	Neuroticism	2.31	0.63	-0.11	0.03	0.01	0.06	0.13	-0.56ˆ***	-0.24ˆ*	-0.42ˆ***	–		
10	Openness to experience	3.89	0.49	0.10	-0.06	0.06	0.01	-0.05	0.24ˆ*	-0.15	0.22ˆ*	-0.21ˆ*	–	
11	Abusive supervision	1.17	0.39	-0.19	-0.03	0.05	-0.20ˆ*	-0.16	-0.07	0.18	0.00	0.13	0.02	–


*^†^*p* ≤ 0.10, ^∗^*p* ≤ 0.05, ^∗∗^*p* ≤ 0.01, ^∗∗∗^*p* < 0.001.*

*^a^Coded 0 = male, 1 = female; ^b^Coded 0 = English, 1 = Dutch.*

*N = 103.*

**Table 3:** Results of the regression analyses for abusive supervision.

	Abusive supervision						
	
	Step 1		Step 2		Without controls		Additional analysis	
				
	*b*	*SE*	*b*	*SE*	*b*	*SE*	*b*	*SE*
Supervisor Age	-0.01	0.01	-0.01	0.01			-0.01	0.01
Language Supervisor Questionnaire							-0.14	0.08
Agreeableness			-0.10	0.10	-0.11	0.10	-0.10	0.10
Conscientiousness			0.23ˆ**	0.09	0.23ˆ**	0.09	0.18ˆ*	0.09
Extraversion			0.03	0.07	0.04	0.07	0.04	0.06
Neuroticism			0.09	0.07	0.10	0.07	0.10	0.07
Openness to experience			0.11	0.08	0.10	0.08	0.10	0.08
*R*^2^	0.04		0.12		0.09		0.14


*Values are unstandardized regression coefficients.*

*^†^*p* ≤ 0.10, ^∗^*p* ≤ 0.05, ^∗∗^*p* ≤ 0.01.*

*^a^Coded 0 = English; 1 = Dutch.*

**N* = 103.*

## Results

### Descriptive Statistics

The means, standard deviations, and correlations are presented in **Table 2**. The correlations reveal that agreeableness, extraversion, neuroticism, and openness to experience are not significantly related to abusive supervision. The correlation between supervisors’ conscientiousness and abusive supervision did not reach significance at *p* < 0.05 (*r* = 0.18, *p* = 0.07).

### Hypotheses Testing

Following Murphy’s (1996) recommendation to study personality using a multivariate framework we conducted regression analyses in which all Big Five personality traits were entered simultaneously (for a similar approach, see Judge and Bono, 2000). The regression analyses revealed that agreeableness, extraversion, neuroticism, and openness are not significantly related to abusive supervision. Supervisors’ conscientiousness was positively related to abusive supervision (see **Table 3**). This indicates that supervisors high in conscientiousness (e.g., those who are well-organized, strongly focused toward achievements, persistent, set high goals for themselves, …), are more likely to be perceived as abusive by their employees. Despite the significant, positive relation between supervisors’ conscientiousness and abusive supervision, it is interesting to note that the Big Five personality traits only explained an additional 8% of the variance in abusive supervision over supervisors’ age. This seems to suggest that, overall, the importance of supervisors’ personality in predicting employees’ experiences of abusive supervision is rather limited.

## General Discussion

Abusive supervision is associated with a wide range of harmful consequences, such as decreased performance and employee wellbeing (see Tepper, 2007), which highlights the importance of preventing the emergence of such devastating workplace behavior. Even though our knowledge on antecedents of abusive supervision has steadily grown during the past years, the research is still scant and little is known about the importance of supervisors’ *personality* in the prevalence of abusive supervision. The present study addressed this issue as we explored whether supervisors’ Big Five personality predicts employees’ experiences of supervisory abuse. While supervisors’ agreeableness, extraversion, openness to experience, and neuroticism were not significantly related to abusive supervision, our findings did reveal a significant, positive relation between supervisor conscientiousness and abusive supervision. Below, we elaborate on the implications and limitations of these findings.

### Theoretical Implications

Our study adds to the existing knowledge on abusive supervision as we explored the influence of supervisors’ Big Five personality in the prevalence of perceived abusive supervision. Even though insights regarding antecedents of abusive supervision have increased steadily during the past years, the majority of research on this topic has investigated the influence of situational – or interpersonal – factors in the emergence of supervisory abuse (e.g., the extent to which supervisors experience fairness themselves; for an overview, see Zhang and Bednall, 2015). Our findings complement this line of research as we focused on intrapersonal rather than interpersonal factors. That is, we focused on supervisors’ *personality* and revealed that the trait of conscientiousness plays a role in the emergence of abusive supervision. As such, our finding that supervisor conscientiousness was positively related to abusive supervision adds to a limited amount of studies showing that supervisor dispositions (e.g., Machiavellianism; see Kiazad et al., 2010) partially explain employees’ experiences of abusive supervision.

Contrary to Tepper’s (2007) predictions and our theoretical expectations, however, neither supervisor agreeableness, nor supervisor neuroticism were significantly related to abusive supervision in our sample. This is somewhat surprising given that supervisors who are tender-minded and kind-hearted (i.e., high in agreeableness) would be more caring toward their subordinates and thus less likely to display abusive supervision. Similarly, given that negative emotions trigger abusive supervision (see for example Hoobler and Hu, 2013), we expected supervisors who are high in neuroticism (and thus more prone to experience frustration and anger) to be particularly susceptible to engage in abusive supervision. Our findings did not support these premises, nor did we find a significant influence of supervisors’ openness to experience or extraversion on subordinates’ experiences of abusive supervision. Although replication studies are highly warranted in order to reach a definitive conclusion regarding the influence of supervisors’ Big Five personality on employees’ perceptions of abusive supervision, for now, it seems appropriate to assume that – apart from conscientiousness – their influence in the prevalence of abusive supervision is negligible.

Previous research may help understand why conscientiousness might have been predictive of abusive supervision. Martocchio and Judge (1997) showed that conscientiousness was related to self-deception leading to think that conscientious individuals have insufficient self-reflection to realize their behavior is inappropriate or even ineffective in their reaction toward employees. Moreover, Witt et al. (2002) revealed that conscientious individuals are difficult to deal with and inflexible. In fact, sufficient reflection and adequate social skills are paramount for conscientious individuals to reach their potential performance. Indeed, Douglas et al. (2004) showed that conscientiousness only predicted performance if those individuals were high on emotional intelligence (see also Witt and Ferris, 2003). This research and our findings, hence, suggest that conscientious individuals with little social awareness or skills are more inclined to adopt behavior that is perceived as abusive. Furthermore, previous work on abusive supervision already highlighted the importance of supervisors’ emotional intelligence toward minimizing employees’ experiences of supervisory abuse. That is, Xiaqi et al. (2012) revealed that supervisors who are better at recognizing others’ emotions and regulating their own emotions are less likely to be perceived as abusive by their employees. In light of this, it would be interesting for future research to examine whether social awareness, emotional intelligence, or social skills not only directly lowers employees’ experiences of abusive supervision, but also might turn around the negative implications of conscientious leaders on their employees. Relatedly, it would be interesting to explore whether specific employee characteristics influence the relation between supervisors’ conscientiousness and employees’ experiences of abusive supervision. For example, given that those high in conscientiousness are oriented toward the achievement of goals, it could well be the case that supervisors high in conscientiousness only display abusive supervision toward those subordinates who hinder the achievement of their goals (e.g., those who perform poorly or are low in conscientiousness themselves).

Finally, our findings show that the influence of supervisors’ personality on employees’ perceptions of leadership clearly depends on the leadership concept under investigation. For example, the meta-analysis of Bono and Judge (2004) showed that supervisors’ extraversion and neuroticism were consistently related to transformational leadership, while the influence of supervisors’ agreeableness, conscientiousness, and openness to experience varied across different studies. Relatedly, Kalshoven et al. (2011) showed that supervisors’ conscientiousness consistently predicted ethical leadership across two studies, while supervisors’ agreeableness, openness to experience and extraversion did not significantly predict employees’ perceptions of ethical leadership in any of these two studies. In our study, we only found a relation between supervisors’ conscientiousness and abusive supervision. As such, these findings clearly show that the importance of specific Big Five personality traits in leadership behavior is largely dependent upon the specific leadership concept under examination. Interestingly, and in contrast with research on leadership emergence and effectiveness (see Judge et al., 2002a), our finding that supervisors’ broad Big Five personality traits only explain a small amount of the variability in employees’ experiences of abusive supervision is similar to their (limited) importance in employees’ perceptions of transformational leadership (Bono and Judge, 2004) and ethical leadership (Kalshoven et al., 2011). These similarities and differences underscore the relevance of exploring the relation between supervisors’ Big Five personality traits and different leadership styles in order to foster our understanding of this matter.

### Practical Implications

Given that abusive supervision is associated with harmful consequences for both organizations and their employees (e.g., lower levels of employee performance, wellbeing, and organizational citizenship behavior; see Tepper, 2007; Martinko et al., 2013), minimizing the occurrence of such instances is essential. Our study adds to the existing knowledge on how organizations can prevent abusive supervision as we showed that of the Big Five personality traits, only conscientiousness significantly predicted employees’ perceptions of supervisory abuse. This finding is particularly interesting given that previous research revealed that highly conscientious persons are often better performers (Barrick and Mount, 1991; Judge et al., 2013) and more likely to emerge as a leader (Judge et al., 2002a). However, promoting such persons into a supervisory position seems to have a drawback as our study indicates that supervisor conscientiousness is positively related to the extent to which employees experience abusive supervision. Therefore, our findings suggest that organizations should take this into consideration when deciding whom to promote into a supervisory position. Additionally, efforts can be undertaken to provide training and mentoring to supervisors. Supervisors may reach a higher awareness on how their actions might be perceived as abusive and develop constructive techniques to improve their interaction with workers. Furthermore, our finding that supervisors’ Big Five personality only explained a limited amount of the variability in employees’ perceptions of abusive supervision indicates that organizations should not limit their efforts to select the ‘right’ people for a supervisory position, but also focus on creating a working environment that minimizes occurrences of abusive supervision (for an overview, see Zhang and Bednall, 2015).

### Limitations and Suggestions for Future Research

Our study has certain limitations which should be discussed in order to allow readers to correctly interpret our findings. First of all, while our study provides insights on the importance of supervisors’ Big Five personality in the prevalence of abusive supervision, we adopted a broad operationalization and did not take lower order facets of each personality trait into account. Previous work, however, has argued that investigating specific facets further adds to the predictability of organizational behavior (see Judge et al., 2008). Indeed, while we found no significant relation between, for example, supervisors’ agreeableness and employees’ perceptions of abusive supervision, it is plausible that subfacets of agreeableness, such as tender-mindedness, are able to predict employees’ experiences of abusive supervision. In light of this, it is interesting to note that Judge et al. (2013) revealed that the subfacets of the Big Five personality traits explained, on average, twice as much variance in employees’ performance than the broad, aggregated traits. This suggests that the use of broad personality traits “may fail to maximize the criterion-related validity of personality by relying on scales that classify people into overly broad personality categories. This is most evident in situations where the narrow facets have differential relationships with the outcomes” (Judge et al., 2013, pp. 891–892). Therefore, we believe it would be interesting for future research to draw on faceted frameworks, such as those of Costa and McCrae (1992) or DeYoung et al. (2007) and explore whether these subfacets explain additional variance in perceived abusive supervision over the more broad operationalization used in the current study.

Our study took a first step to examine the FFM with regard to abusive supervision. Yet, it is theoretically viable that the FFM alone is insufficient to predict abusive supervision and that personality only is triggered under specific situations (Tett and Guterman, 2000), such as perceived autonomy (Barrick and Mount, 1993), job demands (Ng et al., 2008), or unfairness. For example, Skarlicki et al. (1999) revealed that negative affectivity—as an exemplar of neuroticism—as well as agreeableness moderated the effects of fairness on retaliatory behavior in an organizational context. More specifically, people scoring high rather than low on negative affectivity were more severely affected by fairness. Those low in agreeableness were also more influenced by fairness than the more agreeable individuals. Hence, neuroticism and agreeableness may be more likely to predict abusive supervision under conditions in which supervisors felt unfairly treated. Relatedly, our findings revealed a positive relation between supervisors’ conscientiousness and abusive supervision, yet previous research has shown conscientiousness to be beneficial regarding, for example, job satisfaction (Judge et al., 2002b) and individual performance (Judge et al., 2013). As such, it would be interesting for future research to explore moderating variables (e.g., organizational climate; see Patterson et al., 2005) that might mitigate the harmful influence of supervisors’ conscientiousness on abusive supervision.

Secondly, even though the Big Five taxonomy is probably the most used framework to study personality, it is needed to explore whether other personality traits such as the Dark Tetrad (i.e., Machiavellianism, narcissism, psychopathy and everyday sadism; see Paulhus, 2014) are able to explain additional variance in subordinates’ experiences of abusive supervision. Indeed, recent work has highlighted the importance of dark personality traits in understanding interpersonal interactions at work (Schyns, 2015). Kiazad et al. (2010), for example, revealed that supervisors’ Machiavellianism is positively related to abusive supervision. Their finding is particularly interesting given that we observed a *positive* relation between supervisors’ conscientiousness and abusive supervision while previous studies have revealed Machiavellianism to be *negatively* related to conscientiousness (and also to agreeableness; see Paulhus and Williams, 2002; Lee and Ashton, 2005). This could indicate that the observed relations between abusive supervision and supervisors’ conscientiousness (in our study) as well as Machiavellianism (in the study of Kiazad et al., 2010) is not the result of the shared variance between both personality traits, but rather of the variance they uniquely explain in employees’ perceptions of abusive supervision. It would be interesting for future research to pursue this avenue and empirically test whether these dark personality traits indeed explain variance in abusive supervision *above and beyond* more positive personality traits such as the Big Five.

Thirdly, while the purpose of the current manuscript was to shed light on how supervisors’ Big Five personality traits relate to employees’ experiences of abusive supervision, it might be relevant for future research to tune into employees’ personality as well. A study by Grant et al. (2011) indeed showed that the effect of supervisors’ personality might be affected by characteristics of their employees. That is, while several studies have shown that supervisors’ extraversion is positively related to leadership effectiveness, their study revealed that this is only the case when employees are passive and that supervisors’ extraversion even hinders group performance when employees are highly proactive. Interestingly, previous research on abusive supervision revealed that employees’ personality influences employees’ experiences of abusive supervision, because they are more likely to provoke abusive supervision (Henle and Gross, 2014) or to perceive their supervisor’s behavior in such a manner (Brees et al., 2014). As such, it would be interesting for future research to explore whether employees’ personality also affects the relation between supervisors’ Big Five personality traits and employees’ experiences of abusive supervision. For example, while we did not find a significant relation between supervisors’ agreeableness and abusive supervision, it could well be the case that supervisors’ agreeableness does predict abusive supervision when a supervisor is faced with a highly agreeable employee.

Finally, while the movie passage described at the beginning of this manuscript clearly portrays an abusive supervisor, it has to be noted that – in line with Tepper’s (2000) definition – the measure of abusive supervision captures employees’ perceptions of supervisory abuse and not actual supervisory behavior. Indeed, previous research on abusive supervision has outlined that employees’ perceptions of abusive supervision harms employees’ performance, extra-role behavior and wellbeing (Tepper, 2007), yet it remains unclear whether these supervisors actually abuse their employees or are only perceived to do so. In response to this, it has recently been suggested that future research should investigate correlations between perceptions and more objective measures of supervisory abuse (e.g., video data or formal complaints; see Martinko et al., 2013). A recent study by Liang et al. (2015) provide some initial insights in this matter as their experimental study (in which participants were asked to indicate to what extent they would engage in abusive supervision toward their subordinate) and their field study (measuring employees’ perceptions of abusive supervision) revealed similar results. It would be interesting for future research to further explore this avenue in light of our finding. This might advance our insights about whether supervisors’ conscientiousness stimulates actual abusive behavior or only boosts the extent to which employees perceive their supervisor as abusive.

## Conclusion

The harmful consequences of abusive supervision have been widely documented. Although research on antecedents of experienced supervisory abuse has gained increased interest during the past years, little is known regarding the influence of supervisors’ personality in the prevalence of perceived abuse. The present study takes a first step to address this issue as we explored the relation between supervisors’ Big Five personality and employees’ reports of abusive supervision. Our findings revealed that conscientiousness was positively related to abusive supervision, while the overall influence of supervisors’ Big Five personality was rather limited.

## Author Contributions

All authors listed, have made substantial, direct and intellectual contribution to the work, and approved it for publication.

## Conflict of Interest Statement

The authors declare that the research was conducted in the absence of any commercial or financial relationships that could be construed as a potential conflict of interest.
